# Phytoremediation of CYN, MC-LR and ANTX-a from Water by the Submerged Macrophyte *Lemna trisulca*

**DOI:** 10.3390/cells10030699

**Published:** 2021-03-21

**Authors:** Małgorzata Kucała, Michał Saładyga, Ariel Kaminski

**Affiliations:** Metabolomics Laboratory, Faculty of Biochemistry, Biophysics and Biotechnology, Jagiellonian University, Gronostajowa 7, 30-387 Krakow, Poland; kucalagosia@gmail.com (M.K.); michal.saladyga@gmail.com (M.S.)

**Keywords:** anatoxin-a, cylindrospermopsin, microcystin-LR, cyanobacteria, phytoremediation, macrophyte

## Abstract

Cyanotoxins are harmful to aquatic and water-related organisms. In this study, Lemna trisulca was tested as a phytoremediation agent for three common cyanotoxins produced by bloom-forming cyanobacteria. Cocultivation of *L. trisulca* with *Dolichospermum flos-aquae* in BG11 medium caused a release of the intracellular pool of anatoxin-a into the medium and the adsorption of 92% of the toxin by the plant—after 14 days, the total amount of toxin decreased 3.17 times. Cocultivation with *Raphidopsis raciborskii* caused a 2.77-time reduction in the concentration of cylindrospermopsin (CYN) in comparison to the control (62% of the total pool of CYN was associated with the plant). The greatest toxin limitation was noted for cocultivation with *Microcystis aeruginosa*. After two weeks, the microcystin-LR (MC-LR) concentration decreased more than 310 times. The macrophyte also influenced the growth and development of cyanobacteria cells. Overall, 14 days of cocultivation reduced the biomass of *D. flos-aquae*, *M. aeruginosa*, and *R. raciborskii* by 8, 12, and 3 times, and chlorophyll a concentration in comparison to the control decreased by 17.5, 4.3, and 32.6 times, respectively. Additionally, the macrophyte stabilized the electrical conductivity (EC) and pH values of the water and affected the even uptake of cations and anions from the medium. The obtained results indicate the biotechnological potential of *L. trisulca* for limiting the development of harmful cyanobacterial blooms and their toxicity.

## 1. Introduction

Cyanobacteria are one of the most widespread photosynthetic bacteria in the world. They can live in every type of environment and on every type of substrate, and additionally, some of them show the ability to fix atmospheric nitrogen [1,2]. Their presence has been confirmed on land, in freshwater, in saltwater, and in the open air [3,4]. Some cyanobacteria can synthesize secondary metabolites called cyanotoxins, toxic to animals and plants. Out of the many described groups of toxins, three of them are the most commonly determined in freshwater reservoirs. These include hepatotoxic microcystins (MC), cytotoxic cylindrospermopsin (CYN), and neurotoxic anatoxin-a (ANTX-a) [5]. These toxins can pose a public health risk by exposure through skin contact or the ingestion of contaminated drinking water or food [6]. Usually, the concentration of cyanotoxins in the water is minimal and not enough to cause a toxic effect on animals. The situation changes during the mass growth of cyanobacteria able to produce and release cyanotoxins. This state is called cyanobacterial harmful algal bloom (CyanoHAB).

Human activity and environmental changes have led to the accumulation of nutrients in aquatic systems, which has intensified the emergence of CyanoHABs [7,8]. To this day, many physicochemical methods for removing cyanotoxins from water have been confirmed, and most of them, such as activated carbon, UV disinfection, oxidants, and advanced oxidation processes, are used in drinking water treatment [9,10]. Despite the many positive aspects offered by these methods, they require an appropriate infrastructure and qualified personnel and cannot be used everywhere. Phytoremediation can be an alternative or complement to these methods. Usually, higher plants or algae are used for the phytoremediation of heavy metals from water, but some publications have also confirmed their effectiveness in removing cyanotoxins [11,12,13,14]. This is why we are proposing cyanotoxins phytoremediation by *Lemna trisulca* as a natural, environmentally friendly, and easy-to-apply technique to limit the creation and occurrence of CyanoHABs, as well as to limit the concentration of or eliminate the associated toxins. *L. trisulca* in contrast to most of the previously studied plants is a submerged and free-floating macrophyte. This property gives it an advantage over plants floating on the water surface and rooted at the shore of the water reservoir, because this plant still occurs in similar to cyanobacteria ecological niches in the reservoir and is passively moved with them. Additionally, various plants show different responses to cyanotoxins. Some of them seem to be very sensitive to cyanotoxins, such as *Ceratophyllum demersum* [15], and some of them, such as *L. minor* or *Lemna trisulca*, are resistant to higher toxin concentrations than those confirmed in nature [16,17]. Usually, in CyanoHABs, there is a mixture of cyanobacterial species, and the most common freshwater toxic cyanobacteria are *Microcystis*, *Dolichospermum* (*Anabaena*), and *Raphidopsis* (*Cylindrospermopsis*) [18,19].

In our research, we decided to study a two-week cocultivation of cyanobacteria with *L. trisulca*. In our opinion, each method for limiting CyanoHABs should restrict cyanobacterial growth and toxin synthesis within a relatively short time. Moreover, the initial amount of cyanobacteria in the medium in our experiments, which is presented as equivalents of chlorophyll a (chl a, minimum 0.2 mg/L), was much higher than the 0.01 mg/L proposed as a guideline toxin level by the World Health Organization (WHO) and is close to the highest values confirmed in the natural environment [20,21]. *L. trisulca*, in our opinion, is an excellent candidate for cyanotoxin phytoremediation and cyanobacteria elimination, because compared to other studied plants such as *L. minor*, *L. trisulca* is completely submerged in water and occupies similar ecological niches as cyanobacteria. Additionally, currently, green technologies, such as phytoremediation, are constantly developed and researched because they do not cause drastic changes in the environment compared to physicochemical methods [22].

The main aim of this research was to study the potential, effectiveness, and versatility of the common macrophyte *L. trisulca* in not only removing the most common cyanotoxins but also in limiting the CyanoHABs created by the most common freshwater cyanobacterial species, *Microcystis*, *Dolichospermum*, and *Raphidopsis*. According to our best knowledge, there are limited studies about the impact of plants on both cyanobacteria and the toxic compounds produced by them. Little is known about CYN phytoremediation. Within the two-week experiments that we performed, the kinetics of cyanobacteria and plant growth, cyanotoxin contents (in microorganisms cells, the medium, and plant cells), chl a content in all tested organisms, chl b and total carotenoid contents in plant material, pH, conductivity, and selected ion concentration in medium were studied. Our study can be helpful in the “Green Liver Concept” design and application, especially in the case of recreational and drinking water reservoirs, where plants growing near the banks, as well as floating on platforms would reduce their recreational and natural character.

## 2. Materials and Methods

### 2.1. Research Material

The three axenic cultures of the cyanobacteria *Raphidiopsis raciborskii* (*Cylindrospermopsis raciborskii* Woloszynska) strain CS- 505/7 (CSIRO Collection of Living Microalgae), *Dolichospermum flos-aquae* (*Anabaena flos-aquae* Brébisson ex Bornet and Flahault) strain SAG 30.87 (Culture Collection of Algae at Göttingen University), and *Microcystis aeruginosa* (Kützing) strain PCC 7806 (the Pasteur Culture collection of Cyanobacteria) and the axenic macrophyte *Lemna trisulca* (L., laboratory culture) were cultivated separately in BG11 medium [23] in a phytotron at 22 ± 1 °C with 80 ± 5% humidity and 25 µmol photons m^−2^s^−1^ photosynthetically active radiation (PAR) under a 12-h photoperiod (lamps AQUAEL 18 W PLANT). No antibiotics were added to the medium during continuous cultivation, as well as during experiments. All cultures were shaken daily. All cultures after 21 days, under sterile conditions, in a laminar airflow chamber are transferred to fresh medium. 

### 2.2. Experimental Procedure

#### 2.2.1. Preparation of Material

One day before starting the experiment (t_0_), two-week-old cultures of cyanobacteria (6 L) were centrifuged for 10 min at 5000× *g* (Sigma, Osterode am Harz, Germany, 6–16 K Centrifuge, temperature 20 °C) to separate the biomass from the medium, which was removed. Then, 60 mL of fresh BG11 medium was added to the cyanobacterial cells pellets, and afterward, each culture was gently shaken for 24 h on a ChemLand (Stargard Szczeciński, Poland) pendulum shaker at 130 rpm (stock culture) to evenly distribute the cyanobacteria in the medium.

#### 2.2.2. Determination of the Dry Weight of Cyanobacteria in Stock Cultures

Into pre-weighed 2 mL Eppendorf tubes was transferred 1 mL of cyanobacteria stock culture (in three independent replicates for each species). Each whole sample was frozen in liquid nitrogen, lyophilized, and weighed again to determine the dry weight (d.w.) content of cyanobacterial material in the stock medium.

#### 2.2.3. Preparation of Cultures for the Experiments

All experiments were performed in sterile 50 mL Erlenmeyer flasks under similar physiochemical conditions to those described above. In total, 20 mL of fresh BG11 medium was poured into each flask. 

In the first series of experiments, *D. flos-aquae*, *R. raciborskii*, *M. aeruginosa*, and *L. trisulca* were cultivated alone (control). To each flask, the proper volume of stock culture containing the equivalent of 0.7 mg dry weight (d.w.) of cyanobacteria or the initial fresh weight (f.w.) of the entire plant (single polikormone), close to 45 mg (the equivalent of 3.2 mg d.w.) was added. In the second series of experiments, to similarly prepared cultures of cyanobacteria, *L. trisulca* was added—common cultivation. The macrophyte-to-microorganism d.w. ratio was 1:0.22 and it was similar to the average value in a tested natural pond during an algal bloom. 

All cultures were cultivated under similar physiochemical conditions to those described in Section 2.1. and were shaken daily for 10 min. Illustrative photo showing a single set of individual organism cultures or co-cultivation of organisms are included in the supporting material as Figures S4 and S5.

#### 2.2.4. Sample Preparation

Samples (flasks with biomass) were collected at t_0_ and after 1, 4, 7, 11, and 14 days of cultivation. Every time, three independent replicates (three individual flasks) were sampled for each culture. Before proper analysis, all of the flasks were gently shaken for 10 min to mix the material.

For each sample, it was prepared column for separation. At the bottom of a 6 mL glass solid phase extraction (SPE) tube was placed a lyophilized and pre-weighed Whatman™ GF/C glass microfiber filter. The top of each tube was connected to a plastic sieve (0.3 mm mesh diameter). The whole column was placed in a vacuum manifold (Supelco), into which tubes for medium collection were placed. Using the vacuum pump and valve, the underpressure was set at −15 in Hg.

The cultures were poured into the separation columns. The macrophyte remained on the sieve, the cyanobacteria were retained on the microfiber filters, and the medium was collected in the tube. All of the tubes containing medium were closed and transferred for further analysis. The cultivation flasks were rinsed three times with 5 mL of MilliQ water (Millipore, Burlington, MA, USA) for collection and transfer all cyanobacteria cells to the separation column, and afterward, the separation columns were rinsed with 5 mL fresh MilliQ water.

The plants and cyanobacteria collected on the sieves and microfiber filters were frozen in liquid nitrogen and lyophilized. Afterward, the dry weight of the organisms was determined, and the material was placed in the freezer (−27 °C). The separation efficiency has been confirmed microscopically.

#### 2.2.5. Medium Analysis

Right after medium collection, the pH (Mettler Toledo InLab^®^ electrode, Mettler Toledo Five Easy Plus pH/mV meter) and conductivity (EC, HM Digital EC/TDS/TEMP COM-100 conductometer) were measured. In total, 1 mL was transferred to HPLC vials and frozen for further toxin concentration analysis, and an additional 5 mL was frozen for further ion concentration analysis.

#### 2.2.6. Determination of Photosynthetic Pigment Contents

Each lyophilized plant and cyanobacterial sample was mixed with 4 mL of 80% acetone and homogenized on an Omni Sonic Ruptor 400 homogenizer (USA) on ice (3/8” processing tip, gradually increasing the power up to 400 watts, 10 s homogenization). Verification of complete cell lysis was performed microscopically. After the samples were centrifuged for 5 min at 10,000× *g*, 200 µL of the supernatant was transferred to a 96-well plate, and the absorbance at 470, 646, and 665 nm wavelengths was measured on a BioTek Synergy H1 microplate reader. Photosynthetic pigments content was calculated using the equations developed by Wellburn [24]. A total of 1 mL of sample was collected for the analysis of cyanotoxins concentration.

#### 2.2.7. Determination of Toxin Concentrations

To determine the changes in CYN, MC-LR, and ANTX-a concentrations over time, all of the samples were analyzed on the Shimadzu Nexera-I LC-2040C 3D Plus Ultra High-Performance Liquid Chromatograph (UHPLC). The concentration of toxins in the medium was determined directly without any manipulation of the sample. In the case of cyanobacteria and the plant, after measuring the photosynthetic pigments content, 1 mL of the sample (in 80% acetone) was evaporated to dryness and dissolved in 1 mL of 10% methanol. The thus prepared sample was analyzed on UHPLC. The gradient mobile phase consisted of MilliQ water and acetonitrile (both acidified with 0.05% trifluoroacetic acid), in which the organic phase increased from 2 to 90% over 15 min at a flow rate of 0.75 mL·min^−1^. The samples were separated on a Gemini^®^ NX-C18 Column (110 Å, 3.0 µm, 150 mm × 4.6 mm) maintained at 40 °C. The autosampler cooler temperature was 4 °C, and the PDA cell temperature was 40 °C. Toxins were identified by comparing the retention times and UV-spectra determined for commercial standards and quantified by the absorbance at 228, 239, and 261 nm for ANTX-a, MC-LR, and CYN, respectively. A multilevel calibration curve was obtained using commercial standards (from 0.01 to 10.00 µg/mL). Representative chromatograms obtained for samples analysis are included in the supporting material as Figures S1 and S3.

#### 2.2.8. Determination of Ion Concentrations in the Medium

First, 5 mL of medium was collected from each sample, and the concentration of anions (Cl^−^, NO_3_^−^, and SO_4_^2−^) and cations (Ca^2+^, K^+^, Mg^2+^, and Na^+^) were analyzed on the DX600A ion chromatograph (Dionex, Sunnyvale, CA, USA). The medium were filtered through 25TF PURADISC™ membrane filters (Whatman, Little Chalfont, UK) with a diameter of 0.45 µm. Using the AS40 autosampler, solutions were introduced into IonPac analytical columns. The AS9-HC column (4 × 250 mm) and a liquid phase consisting of a 9 mM solution of Na_2_CO_3_ were used for the determination of anions. The concentrations of the cations were determined using a CS-12A column (4 × 250 mm) with 20 mM methanesulfonic acid (MSA, Sigma–Aldrich, Munich, Germany) as the eluent. The flow rate of the eluent through the column in both cases was 1 mL/min. The separated ions were identified by the ED50 detector, and the ion concentrations were analyzed using the PeakNet software (Dionex, USA).

#### 2.2.9. Chemicals

ANTX-a, CYN, and MC-LR standards were purchased from LGC Standards company (Teddington, UK), and the chl a standard, Whatman™ GF/C glass microfiber filters, vacuum manifold and solvents for UHPLC analysis were acquired from Merck (Germany).

#### 2.2.10. Statistical Analysis

The data from each experiment are reported as the mean (±s.d.) of three independent replicates. All results were subjected to ANOVA and Student’s *t*-test. Individual means were compared for significant differences at *p* < 0.05.

## 3. Results and Discussion

Water eutrophication and increased CyanoHABs formation are two of the largest problems for aquatic environments. Many physicochemical and mechanical methods have been created and developed to prevent, mitigate, and eliminate cyanobacteria and their toxins from water bodies [6,25]. All these methods eliminate toxins and cyanobacterial cells within a relatively short time but require the appropriate infrastructure and investment of money. This is why most methods, such as advanced oxidation processes, water filtration, or the artificial mixing of the water column are used only in strategic drinking water tanks in developed economic regions [26,27]. On the other hand, there are natural processes such as phytoremediation, adsorption onto sediments, and photolysis which require more time than physiochemical methods but are easier to apply and environmentally friendly and do not involve high costs [13,22,28]. The perfect plant for phytoremediation should demonstrate adsorption and degradation of toxic compounds, be resistant to high toxin concentrations, not be toxic to the water reservoir and related fauna, and not only mitigate or eliminate toxins but also prevent their synthesis by other organisms. *L. trisulca* fulfills all of these functions, and its occurrence has been confirmed in many countries where CyanoHABs occur. This macrophyte has previously been shown to be able to degrade ANTX-a, resistant to toxin high concentrations, and prevent the growth of cyanobacteria [13,28]. In another study, the potential of *L. minor* to bioaccumulate and reduce the MC-LR concentration in raw lake surface water was confirmed [12]. To this day, there is limited information about the phytoremediation of CYN by plants. Some reports have investigated the effects or accumulation of CYN in vegetables belonging to the *Apiaceae* (parsley, carrot), *Asteraceae* (lettuce), *Brassicaceae* (mustard plant), and *Fabaceae* (common bean, pea) families [29,30,31,32]. This information is important to farmers or companies irrigating their fields with water from lakes, but the phytoremediation factor of this plant for CYN removal from water is insignificant. Another study on *Azolla filiculoides* showed a drastic decrease in growth and 99.8% inhibition of protein synthesis at 5 µg CYN·mL^−1^ [33]. In the same paper, a low uptake of CYN (1.314 µg·g^−1^ f.w.) was determined, and the authors concluded that the tested fern is not suitable for CYN phytoremediation. The studied *L. minor* showed sufficient defense mechanisms against CYN, but at the same time, this toxin promotes oxidative stress at concentrations of 2.5 or 25.0 µg·L^−1^. In our studies, *L. trisulca* did not cause significant changes in biomass accumulation or the contents of photosynthetic pigments and proteins, nor in photosynthesis or respiration processes at purified CYN concentrations < 5 µg mL^−1^ (data in publishing). Based on these results, in this study, we decided to verify its potential to mitigate or eliminate CyanoHABs created by *R. raciborskii, D. flos-aquae*, and *M. aeruginosa* and the macrophyte potential to eliminate CYN, MC-LR, and ANTX-a synthesized by these cyanobacteria. 

### 3.1. Biomass Accumulation

**Figure 1:**
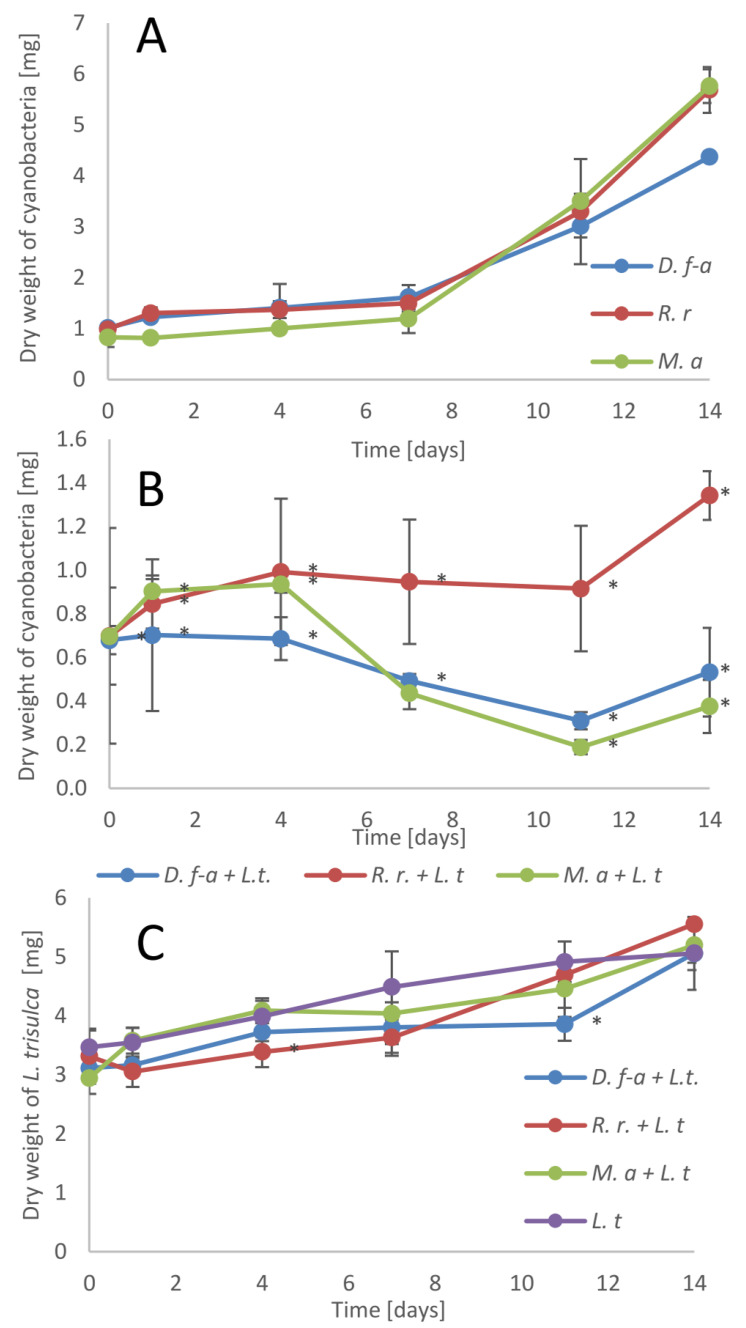
Kinetic of biomass accumulation of cyanobacteria cultivated separately (**A**) or with the addition of the macrophyte (**B**) and *L. trisulca* (**C**)*. D.f-a* corresponds to *D. flos-aquae*, *R.r—R. raciborskii*, *M.a—M. aeruginosa*, *L.t—L. trisulca*, n = 3 ± s.d. * means significant differences at *p* < 0.05 compared to control.

In all experiments, we used living cyanobacterial cells to study plant-microorganism interactions. First, we measured the biomass accumulation in cyanobacteria and plant cultivation alone and together. Cyanobacteria cultivated without the macrophyte demonstrated a good increase in biomass within 14 days (Figure 1A).

During the first 5 to 7 days, acclimatization to the fresh medium was observed, and subsequently, the constant biomass accumulation of all tested cyanobacteria cultures was indicated. After two weeks, the cyanobacterial biomass was from 4.3 times higher for *D. flos-aquae* to 6.9 times higher for *M. aeruginosa* in comparison to the initial weight. Similar results were obtained by other authors for various cyanobacterial species under optimized growth conditions [34,35]. The addition of the macrophyte significantly reduced biomass accumulation by cyanobacteria in all tested samples (Figure 1B). Within two weeks of cocultivation, the biomass of *M. aeruginosa* was reduced by 47% in comparison to the t_0_ value and was 12 times lower in comparison to the control sample (cultivated without macrophyte). For *D. flos-aquae*, the corresponding values were 21% and 8.2 times. Only *R. raciborskii* increased its initial biomass by 1.9 times during the 14-day cocultivation with the macrophyte, but in comparison to the control, this value was reduced 3 times. *L. trisulca* cultivated alone and together with cyanobacteria showed a similar rate of biomass accumulation, and after two weeks, its mass was, on average, higher by 163% in comparison to the initial value (Figure 1C). These results confirm the hypothesis that the presence of *L. trisulca* limits the growth of cyanobacteria. This may be due to: (1) higher nutrient absorption rate by the plant [36], (2) the effect of shading the plant leaves over the culture, reducing the availability of photosynthetic radiation, (3) plant secretion of allelopathic compounds, (4) attachment of some cyanobacteria cells to the plant. The obtained results are similar to those obtained for the *L. trisulca* and *D. flos-aquae* interaction, in which the living organisms and total medium volume ratio was much higher [28]. This relationship was also demonstrated in another work wherein the cooperation between three submerged plants (*Lindernia rotundifolia*, *Hygrophila stricta*, and *Cryptocoryne crispatula*) revealed the inhibitory effects on cyanobacterial growth under natural conditions in the raw water of Guishui Lake [37]. 

### 3.2. Photosynthetic Pigments

**Figure 2:**
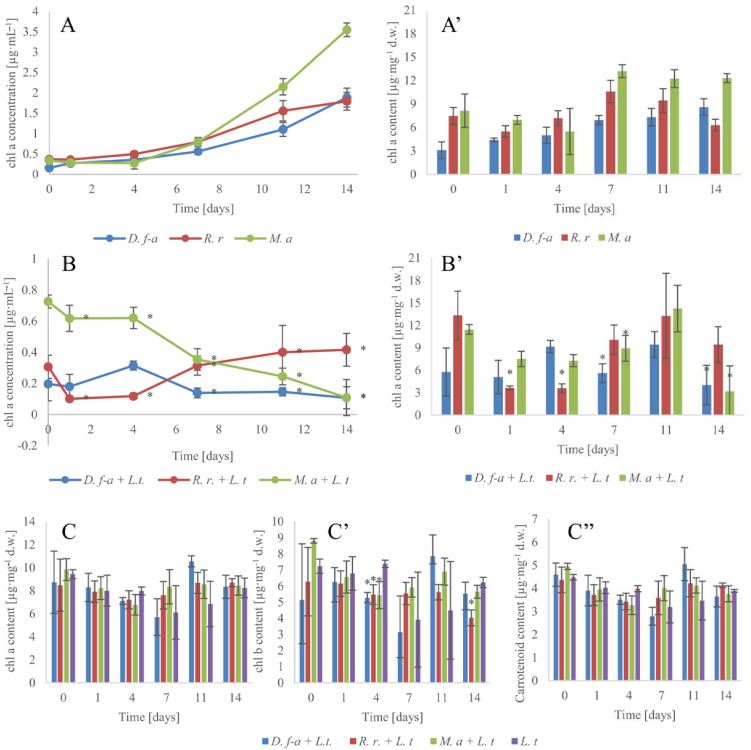
Chl a concentration in samples with cyanobacteria cultivated alone (**A**) or co-cultivated with the macrophyte (**B**). Chl a content in cyanobacterial cells cultivated alone (**A’**) and with the addition of *L. trisulca* (**B’**). Photosynthetic pigment contents in *L. trisulca:* chl a (**C**), chl b (**C’**), and total carotenoid (**C’’**) contents cultivated separately (*L.t*, control) or with the addition of cyanobacteria, n = 3 ± s.d., * means significant differences at *p* < 0.05 compared to control.

In the environment, the intensity of cyanobacterial blooms and associated health risks, according to WHO (2003) [38], is measured as the number of individual cyanobacterial cells·mL^−1^ or concentration of chl a·L^−1^. Among the three tested cyanobacterial species, only *M. aeruginosa* is unicellular and allows easy calculation of the number of cells, while the two remaining species are filamentous, and without proper separation techniques, the counting of individual cells is complicated [39]. This is why in our study, we decided to measure the condition of our cultures by measuring the biomass and concentration of chl a. The chl a concentration (Figure 2A,B) corresponds to changes in cyanobacterial biomass. During the adaptation phase (up to 7 days), in the case of individual cyanobacterial species cultivation, the changes in the chl a concentration were insignificant. From 7 to 14 days of the experiment, an increased concentration of this dye and increased total and per mg dry weights of cyanobacterial cells (A’) were observed. Most likely, in the fresh medium, the lack of stressors such as too-dense culturing promotes the synthesis of this dye, especially in *M. aeruginosa* cells (B’). Similar changes were obtained for *Nostos* sp., in which the combined effect of the irradiance, pH, and inorganic carbon availability effects the photosynthetic pigment content and photosynthesis within 4 days [40].

In the case of cyanobacteria co-cultivated with the plant, decreases of the chl a concentration in *M. aeruginosa* and *D. flos-aquae* samples from the first day were noted, and the stabilization of its concentration in the *R. raciborskii* sample was observed within the 14-day experiment (Figure 2B). Similar chl a contents over time in cyanobacteria cells prove the gradual inhibition of their proliferation by the plant. This is important, because the application of physical or chemical methods such as algicides to cyanobacteria can destroy their cells immediately but, at the same time, release a great amount of cyanotoxins into the water [41]. The chl a analysis corresponded to the dry weight measurements and confirmed the fast but safe action and the possibility of using *L. trisulca* for the reduction in cyanobacterial blooms. 

Additionally, the cocultivation of *L. trisulca* with any cyanobacterial species did not affect the chl a (Figure 2C), chl b (Figure 2C’), and total carotenoid contents (Figure 2C’’) in the plant tissues in comparison to the control. These results confirm and extend the current knowledge on the resistance of this macrophyte to cyanotoxins [17].

### 3.3. Water EC and pH

**Figure 3:**
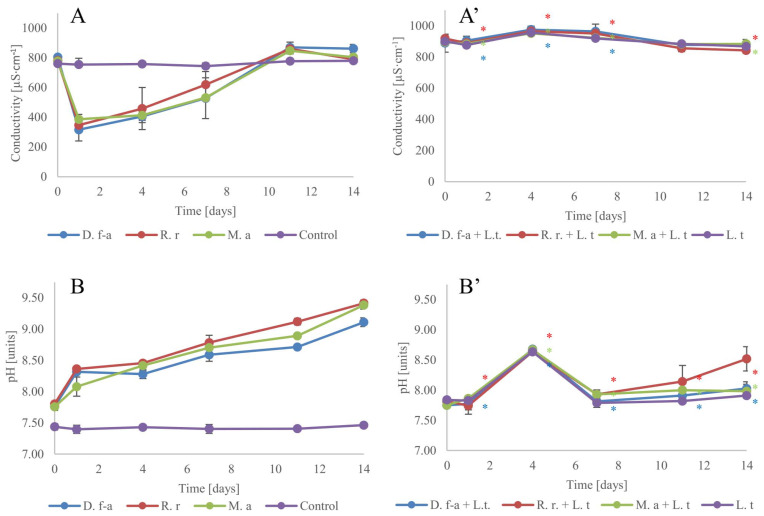
Electrical conductivity (EC) of medium in which cyanobacteria were cultivated alone (**A**) or co-cultivated with the macrophyte (**A’**), pH of the control samples (**B**, cultivation without macrophyte, or pure nutrient medium) and the experimental samples (**B’**), n = 3 ± s.d., * means significant differences at *p* < 0.05 compared to control.

The operational monitoring of source water according to WHO guidelines includes the measurement of its, inter alia, algal growth, color, pH, and conductivity [42]. The required ranges of these values are defined in the relevant legal acts, and in the case of conductivity, it is usually <1000 µS·cm^−1^. It was shown that a high conductivity (1500 µS·cm^−1^) had a neutral or positive effect on growth for the *R. raciborskii* strain [43]. In other papers, it was shown that the water conductivity affected the abundance of selected microorganisms in both freshwater and seawater [44,45]. In our experiments, the initial conductivity of the medium was 760 µS·cm^−1^, and without the addition of any living organism within 14 days it was stable (Figure 3A). The cultivation of cyanobacteria in a medium within the first 24 h radically decreased its conductivity, which was associated with an accumulation of cyanobacterial cell-accessible ions. From the first to 11th day of cultivation, the medium conductivity gradually increased. It was adequate for the gradual release of organic compounds into the medium. The addition of *L. trisulca* to all analyzed cultures stabilized the medium conductivity. The absorption of ions from the medium was balanced by the release of secondary metabolites by the organisms. These experiments confirm environmental observations indicating that macrophytes are improving the water quality in shallow eutrophic lakes by nutrient accumulation, and in macrophyte-dominant ponds, the conductivity is generally relatively low [46,47]. 

The influence of cyanobacteria on pH values has been well known and studied for many years [48]. These microorganisms contain a CO_2_-concentrating mechanism (CCM), which is pH-dependent. Under alkaline conditions, the CCM mechanism is more energetically efficient [49]. This property and the adsorption carbon dioxide from water in the form of carbonic acid are mainly responsible for raising the pH value in water. In our experiments, the pH was measured at the same time (after 3 h of irradiation). All analyzed cyanobacteria species increased the medium pH values (Figure 3B). For *M. aeruginosa* and *R. raciborskii*, after 14 days of cultivation, the pH increased by an average of 1.6 units. In the case of *D. flos-aquae*, the mean change was 1.4 units. Cocultivation of cyanobacteria and the macrophyte caused similar changes in the pH in all analyzed species (Figure 3B’). On the fourth day of the experiment, an increase in the pH by an average of 0.9 units was noted, followed by its decline and stabilization. Only *R. raciborskii* on the last day of the experiment increased the pH by 0.7 units (in other cases it was 0.25 units). 

### 3.4. Ion Uptake

**Figure 4:**
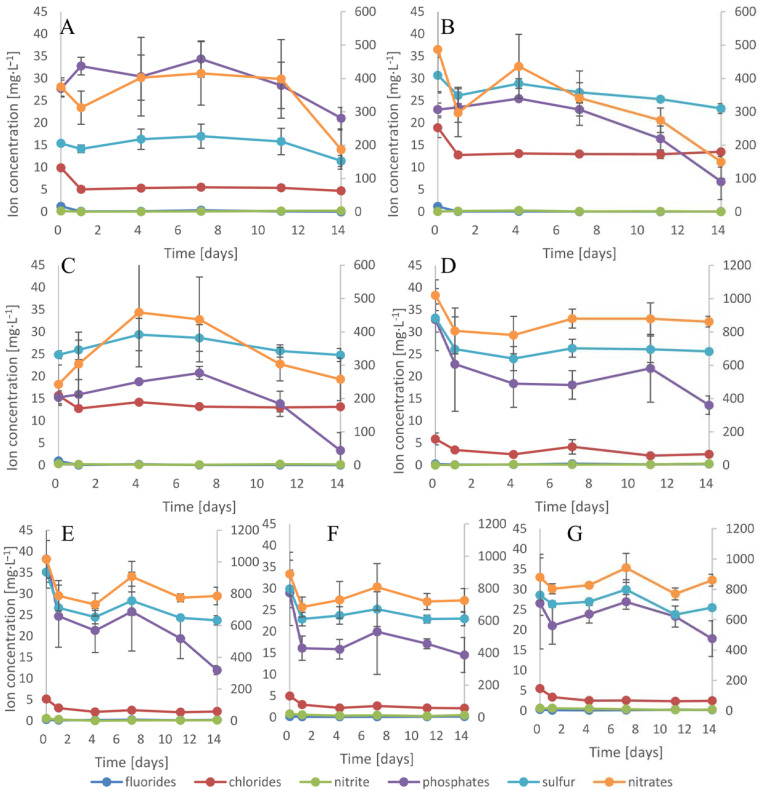
Anion concentrations in medium with *D. flos-aquae* (**A**), *M. aeruginosa* (**B**), *R. raciborskii* (**C**), *L. trisulca* (**D**) cultivated alone or cocultivated with the macrophyte (**E**,**F**,**G**, respectively). The secondary axis specifies the concentration of nitrates n = 3 ± s.d.

**Figure 5:**
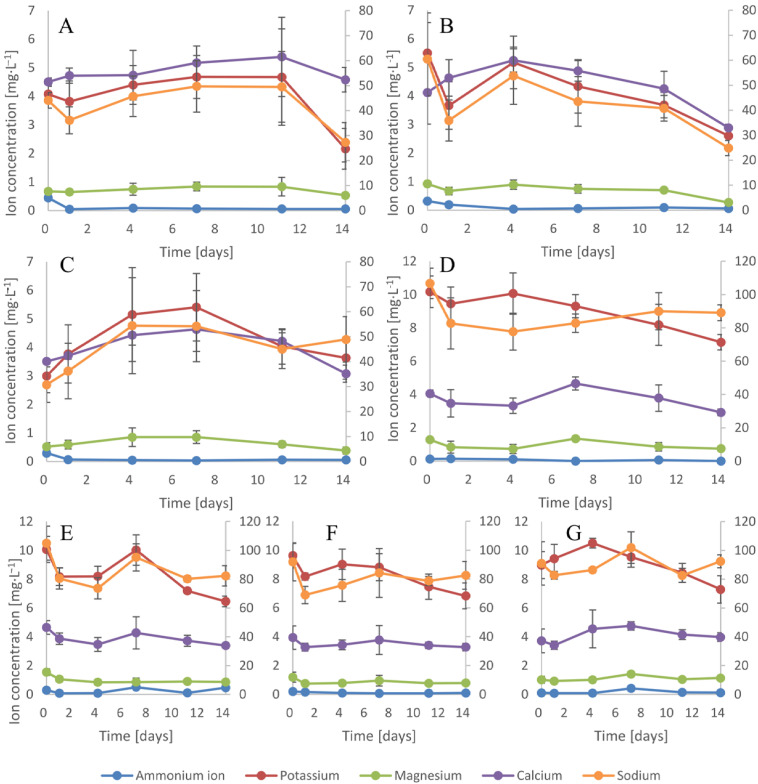
Cation concentrations in medium with *D. flos-aquae* (**A**), *M. aeruginosa* (**B**), *R. raciborskii* (**C**), *L. trisulca* (**D**) cultivated alone or co-cultivated with the macrophyte (**E**,**F**,**G**, respectively). The secondary axis specifies the concentration of sodium, n = 3 ± s.d.

The main impacts on the medium EC and its pH involve dissolved ions. Cyanobacteria and plants, as autotrophic organisms, require specific, essential ions for proper growth and development. Both organisms compete with each other for similar chemicals. Additionally, some of the macrophytes show resistance to high concentrations of ions and various organic or inorganic pollutants present in water [50]. These plants are used in the phytoremediation or bioremediation of polluted water. Among the various aquatic plant species, *Lemna* is one of the most effective macrophytes that have been applied in phytoremediation studies [51]. In this manuscript, we studied the impacts of cyanobacteria and plants cultivated alone and together on the ion concentration and composition (Figure 4 and Figure 5). Among all nutrients, nitrogen is the most imperative element for proper growth and development for both cyanobacteria and plants. All plants utilize nitrogen in the form of NO_3_^-^ and NH_4_^+^, and additionally, some cyanobacteria species such as, inter alia, *Anabaena*, *Nostoc*, and *Cylindrospermopsis* demonstrate the ability to store nitrogen inside cells and are capable of nitrogen fixation [52]. Nitrogen present in the form of nitrates constituted the main pool of this element in the medium. Within 14 days, the single cultivation of *D. flos-aquae* adsorbed 50% of the initial amount of nitrates, while *R. raciborskii* absorbed 69%, *M. aeruginosa* absorbed 6%, and *L. trisulca* absorbed 16% (Figure 4A, Figure 4B, Figure 4C, and Figure 4D, respectively). During the cocultivation experiments, nitrates were taken up mainly during the first 24 h, and afterward, the nitrogen concentration was relatively constant. Finally, in the medium, the range from 3% of the initial concentration of nitrates in *M. aeruginosa/L. trisulca* cultivation to 22% in the *D. flos-aquae/L. trisulca* cocultivation was detected (for *R. raciborskii/L. trisulca*, it was 19%). When the concentration of N in water is high and its ratio to phosphorus in the form of phosphates is also high, phosphorus controls the growth rate. The average initial N:P ratio in our experiments was 25:1, and this promoted the creation of algal blooms. The phosphate concentrations in single cultivations decreased by 24, 70, 78, and 59% within 14 days for *D. flos-aquae*, *R. raciborskii*, *M. aeruginosa*, and *L. trisulca*, respectively. During cocultivation, the concentration of this anion decreased by 69, 50, and 33%, respectively (Figure 4E–G). Moreover, during cocultivation, a leading influence of the macrophyte on the amount and ratio of anions taken up was observed. 

It has been shown that the concentration of divalent cations such as (Mg^2+^, and Ca^2+^) in the environment has a significant influence on cyanobacterial scum formation by some *M. aeruginosa* strains [53]. In our study, the presence of the macrophyte in the cocultivation experiments affected the stabilization of the cation concentration in the medium (Figure 5). Moreover, no cyanobacterial scum formation was observed in the cultures. 

### 3.5. Cyanotoxin Concentration

**Figure 6:**
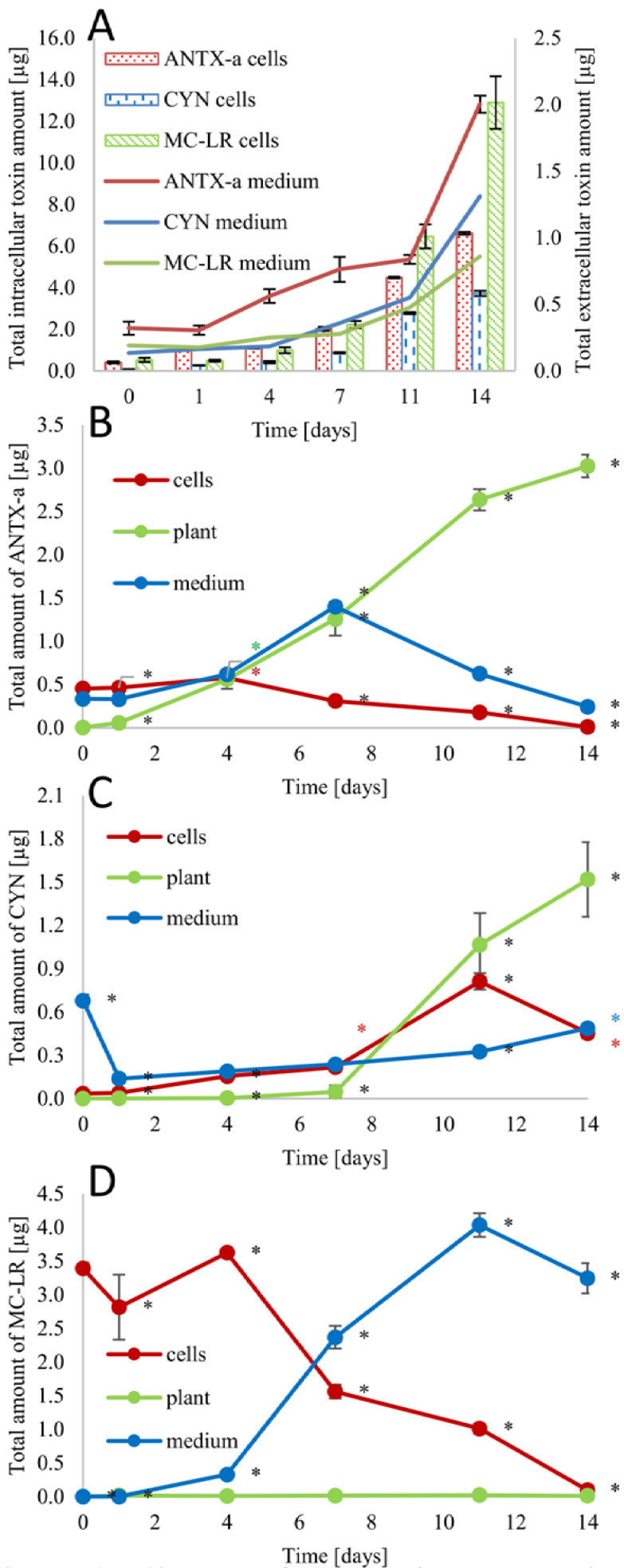
Changes of cyanotoxin contents in cyanobacteria cultivated separately (**A**, intracellular - bars, extracellular - lines) or with the addition of macrophyte to the culture of *D. flos-aquae* (**B**), *R. raciborskii* (**C**), and *M. aeruginosa* (**D**, extracellular, intracellular, and adsorbed/attached to plant), n = 3 ± s.d., * means significant differences at *p* < 0.05 compared to control.

Each cyanobacterial bloom can cause water quality problems, such as increased turbidity or oxygen depletion, resulting in the death of aquatic fauna and flora. Additionally, some cyanobacteria blooms are dominated by species capable of producing toxins, which further increases the risk associated with blooms [54]. This is why in our research, we used the three most common toxic freshwater cyanobacteria species. To simplify the calculations in our experiments, we determined the total intracellular and extracellular toxin contents (Figure S6 present cyanotoxins content calculated for mg of cyanobacterial dry weight). Under optimal conditions, in single cultivations of cyanobacteria, an increase in the total amount of determined toxins was observed (Figure 6). This was related to the increase in the number of cyanobacteria cells and their biomass (for comparison Figure 1). Within 14 days, the total amount of toxins (intra and extracellular) increased by 11.6 times for ANTX-a, by 19.2 times for MC-LR and by 23.9 times for CYN. Similar results were demonstrated for CYN synthesized by *Aphanizomenon ovalisporum*, for which over 9 days, the total amount of CYN increased more than four times [55]. The ratio of the intracellular to extracellular pool of toxins in the case of single cyanobacterial cultivation grew from 1.3 to 3.3 for ANTX-a, from 0.6 to 2.8 for CYN, and from 2.8 to 15.0 for MC-LR. A massive release of cyanotoxins into the water usually occurs during cell death and lysis but can also be the result of allelopathy or a relatively sudden stress factor [6]. The obtained data (biomass accumulation and toxin amount and ratio) confirmed that the single cultivation of cyanobacteria within 14 days promotes their growth and mass reproduction. 

Cocultivation of *L. trisulca* with *D. flos-aquae* caused reducing the number of cyanobacterial cells in the medium, the release of the intracellular pool of ANTX-a, and the adsorption of the toxin by the plant. In comparison to the control sample, after 14 days, the total amount of toxin decreased 3.17 times. A significant amount of macrophyte-related toxin (92%) may also be caused by the attachment of cyanobacteria cells to the macrophyte. In the case of cocultivation of *L. trisulca* with *R. raciborskii*, a 2.77-times smaller concentration of CYN was detected in comparison to the control sample. Moreover, after 2 weeks of cultivation, 62% of the total pool of CYN was associated with the plant. This was probably due to the binding of *R. raciborskii* cells to the macrophyte. Cocultivation of the plant and *M. aeruginosa* caused the greatest decrease in total toxin amount. After 14 days, a slightly lower total concentration of MC-LR was determined than at t_0_, and in comparison to the control sample, the concentration decreased more than 310 times. *L. trisulca* did not adsorb or attach to MC-LR during the experiment. In the last case, it was demonstrated that with cyanobacterial cell death and lysis, the intracellular pool of MC-LR was released into the medium. The attachment of cyanobacteria cells to the macrophyte has also not been demonstrated. In other works, it was confirmed that *L. trisulca* also demonstrated the ability to degrade adsorbed ANTX-a [13], and another species of *Lemna—L. minor* exhibited the ability to eliminate MC-LR from water enriched with it, and *L. gibba* showed MC-LR accumulation from toxic *Microcystis* culture extract [12,56,57]. In comparison to these two toxins, little is known about CYN phytoremediation by macrophytes, and current studies are mainly based on the biodegradation of this compound by microorganisms such as *Aeromonas* sp. [58]. In comparison to physical or chemical processes, the release of the intracellular pool of cyanotoxin into water is violent, which leads to a rapid increase in their concentration in the water and may lead to poisoning of other organisms, while plants work by gradually removing cyanobacterial cells and releasing the toxins. Additionally, the small amounts of toxins released into the water by plants undergo natural decomposition (under certain conditions), bind to the sediment, or become an energy source for microorganisms or other plants [57,58,59,60]. 

## 4. Conclusions

Using plants to improve water quality and restrict or remove CyanoHABs is not as fast as chemical or physical processes, but in comparison, it is less expensive, is more environmentally friendly, and can be used without applying advanced technology. In our experiment, we confirmed that one of the most popular submerged freshwater macrophytes, *L. trisulca*, not only stabilizes the EC and pH values of medium and affects the even uptake of cations and anions from the medium but can also prevent the formation of or cause the safe elimination of CyanoHABs. It limited the growth and development of three toxic cyanobacteria, *D. flos-aquae*, *R. raciborskii*, and *M. aeruginosa*, by limiting their biomass accumulation and significantly restricted the amount of toxins that they synthesized. *L. trisulca* also showed the ability to absorb or attach ANTX-a and CYN and appropriate cyanobacteria that synthesize them, and to transfer the intracellular pool of MC-LR extracellularly. Based on the obtained results, this plant seems to be an excellent candidate for practical use in water treatment plants based on the “Green Liver Concept”.

## Data Availability

The data presented in this study are available on request from the corresponding author until they are entered into the open repository of the Jagiellonian University (ruj.uj.edu.pl).

## Supplementary Materials

The following are available online at https://www.mdpi.com/2073-4409/10/3/699/s1, Figure S1: Representative chromatograms obtained for samples with ANTX-a, Figure S2: Representative chromatograms obtained for samples with MC-LR, Figure S3: Representative chromatograms obtained for samples with CYN, Figure S4: Illustrative photo showing a single set of individual organism cultures, Figure S5: Illustrative photo showing co-cultivation of organisms, Figure S6: Cyanotoxins content calculated for mg of cyanobacterial dry weight.
