# Supplementary material for: Phytoremediation of CYN, MC-LR and ANTX-a from Water by the Submerged Macrophyte Lemna trisulca
Source: Cells. 2021 Mar 21;10(3):699. doi: 10.3390/cells10030699 (PMC8004190; doi:10.3390/cells10030699)
Supplement: Supplementary file 1 [file cells-10-00699-s001.pdf]

Supplementary materials

Cyanotoxins were identified by comparing the retention times and UV-spectra determined for commercial standards and quantified by the absorbance at 228, 239, and 261 nm for ANTX-a (Fig. 1), MC-LR (Fig. 2), and CYN (Fig. 3), respectively.

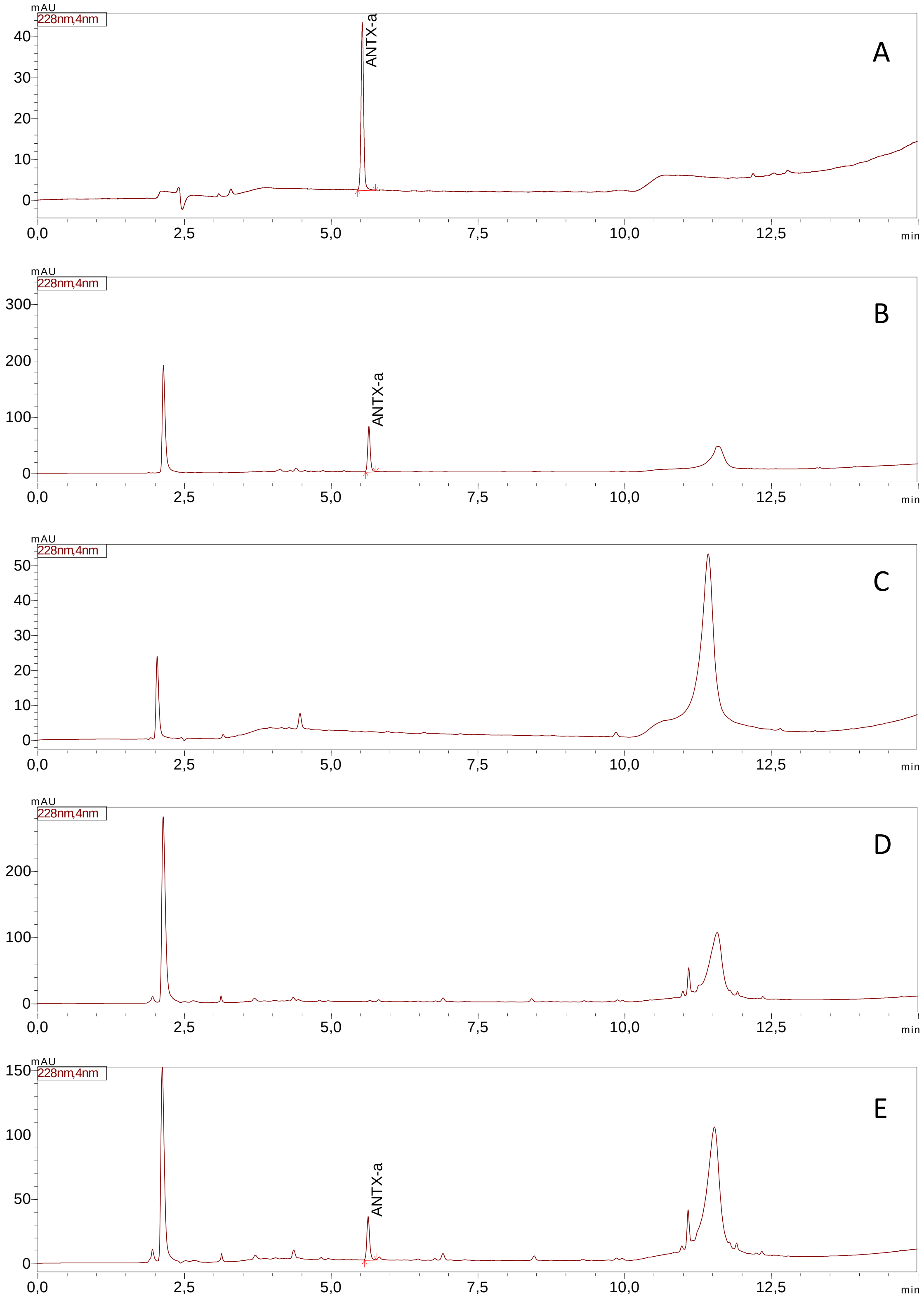

Figure S1. Representative chromatograms obtained for samples with ANTX-a. Chromatogram of: commercial standard of ANTX-a (A), 10% methanol extract from *D. flos-aquae* after 14 days separately cultivation(B), or co cultivated with *L. trisulca* (C), 10% methanol extract from *L. trisulca* cultivated alone by 14 days (D), 10% methanol extract from *L. trisulca* co-cultivated 14 days with *D. flos-aquae* (E)

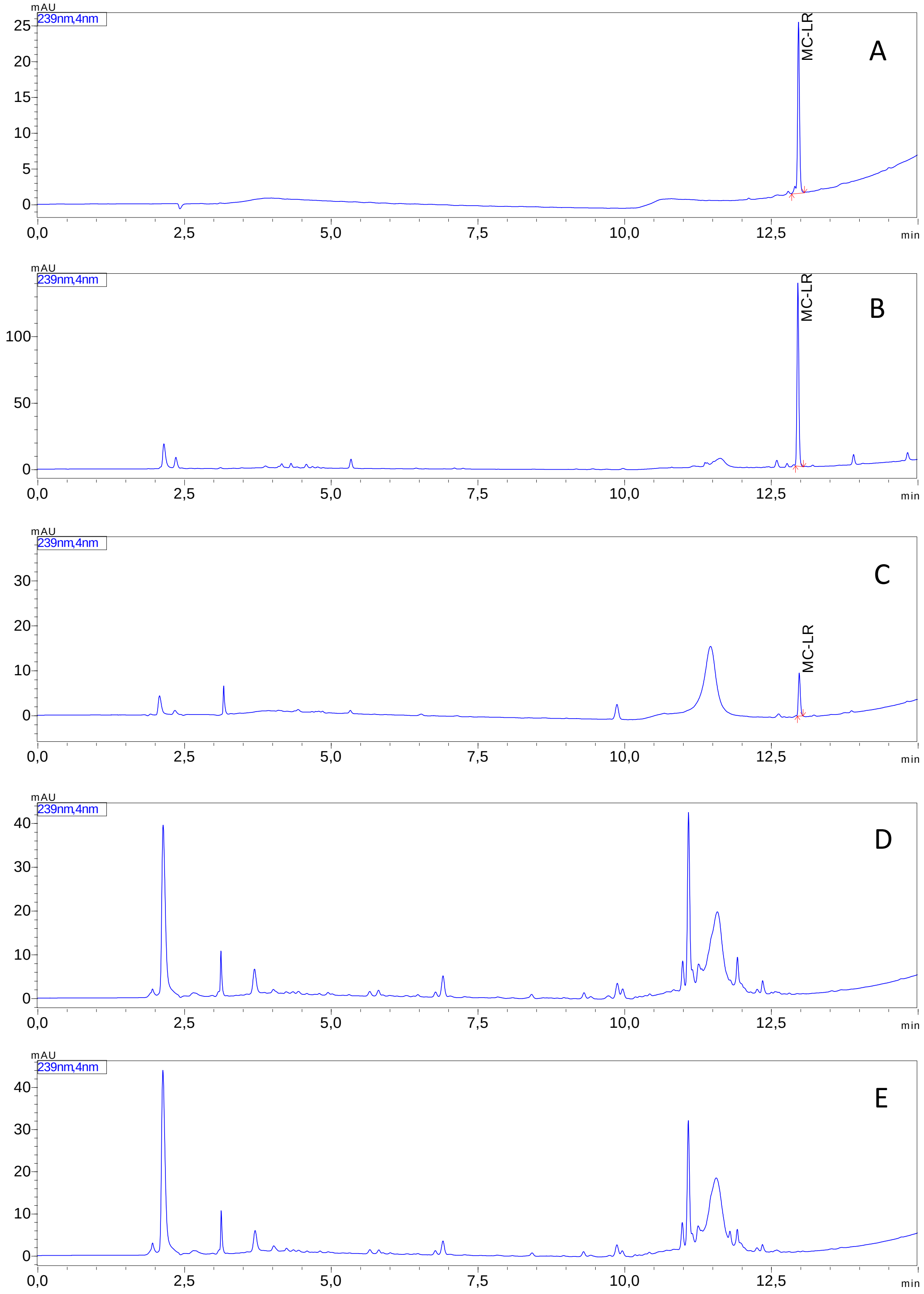

Figure S2. Chromatogram of the commercial standard of MC-LR (A), 10% methanol extract from *M. aeruginosa* after 14 days separately cultivation(B), or co-cultivated with *L. trisulca* (C), 10% methanol extract from *L. trisulca* cultivated alone by 14 days (D), 10% methanol extract from *L. trisulca* co-cultivated 14 days with *M. aeruginosa* (E)

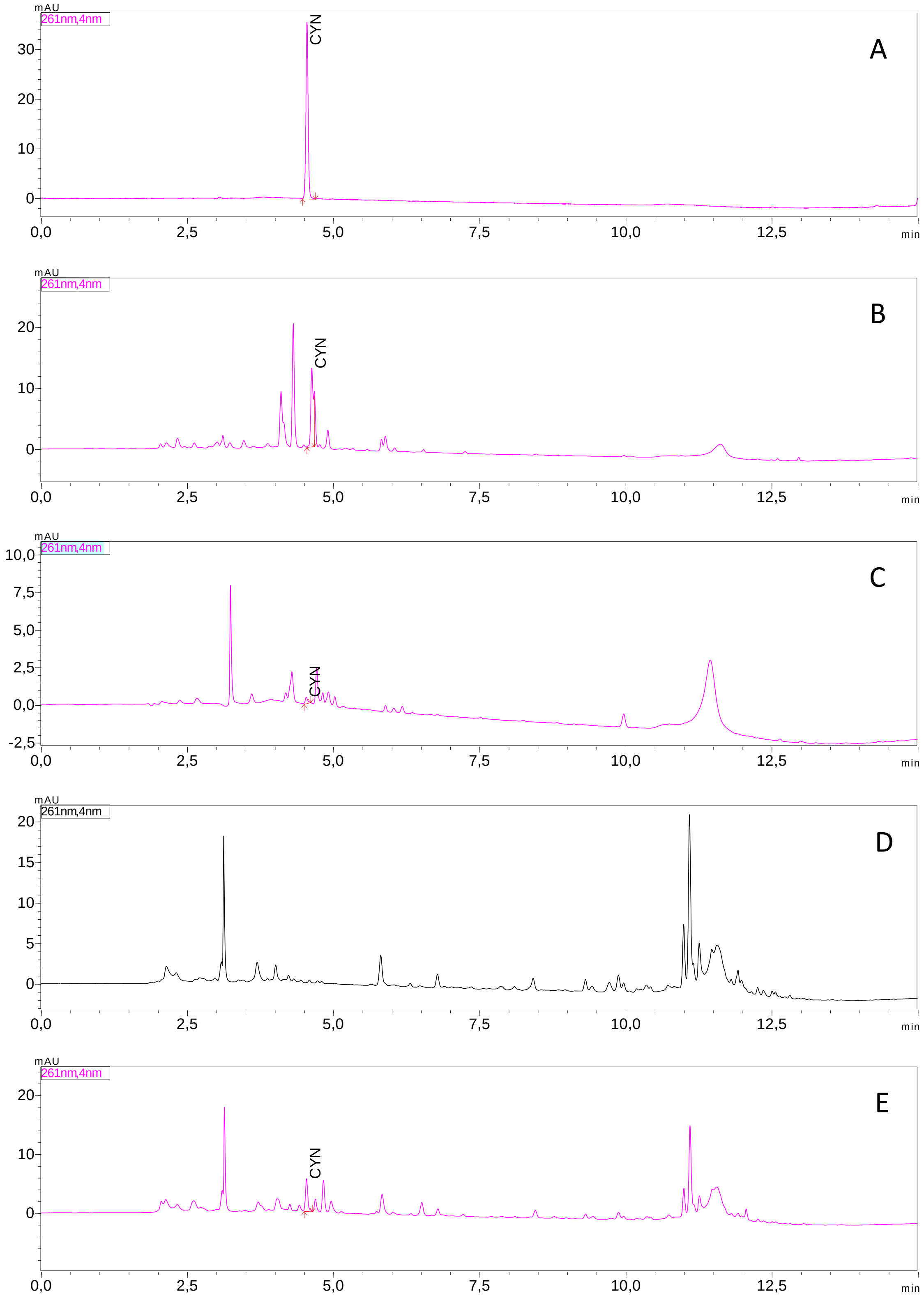

Figure S3. Chromatogram of CYN standard (A), 10% methanol extract from *R. raciborskii* after 14 days separately cultivation (B), or co-cultivated with *L. trisulca* (C), 10% methanol extract from *L. trisulca* cultivated alone by 14 days (D), 10% methanol extract from *L. trisulca* co-cultivated 14 days with *R. raciborskii* (E)

Sample preparation:

All experiments were performed in sterile 50 mL Erlenmeyer flasks. One whole flask with pure medium, or medium with organisms was a single sample. In total, 20 mL of fresh BG11 medium was poured into each flask. In the first series of experiments, *D. flos-aquae*, *R. raciborskii*, *M. aeruginosa*, and *L. trisulca* were cultivated alone (Fig. 4). To each flask, the proper volume of stock culture containing the equivalent of 0.7 mg dry weight (d.w.) of cyanobacteria or the initial fresh weight (f.w.) of the entire plant (single polikormone), close to 45 mg (the equivalent of 3.2 mg d.w.) was added. In total each sample was prepared in 18 replicates (3 independent repetitions for 6 sampling days)

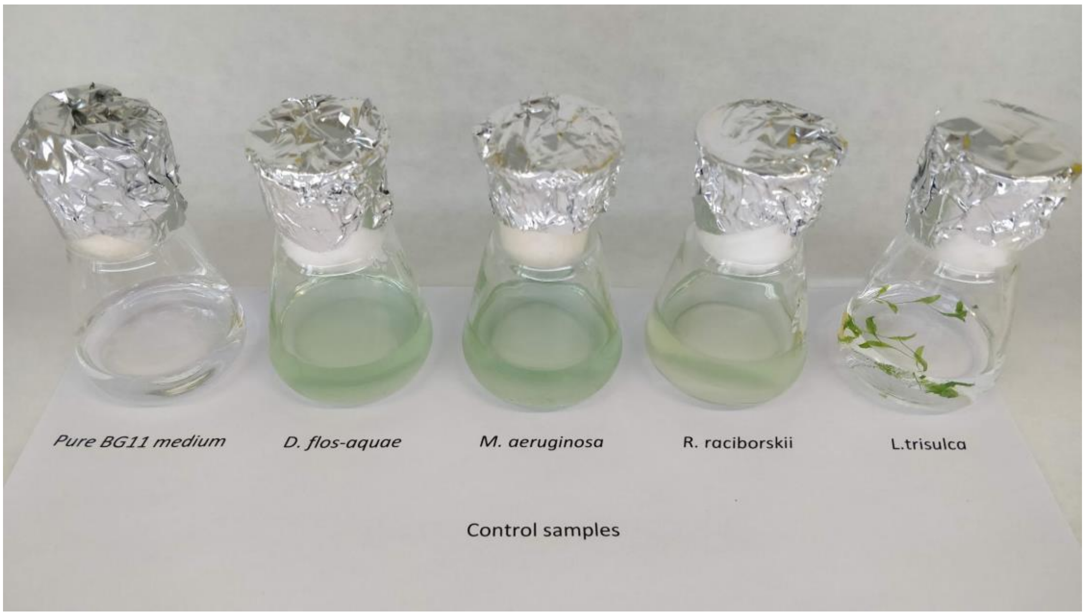

Figure S4. Illustrative photo showing a single set of individual organism cultures

In the second series of experiments, to similarly prepared cultures of cyanobacteria, *L. trisulca* was added - common cultivation (Fig. 5)

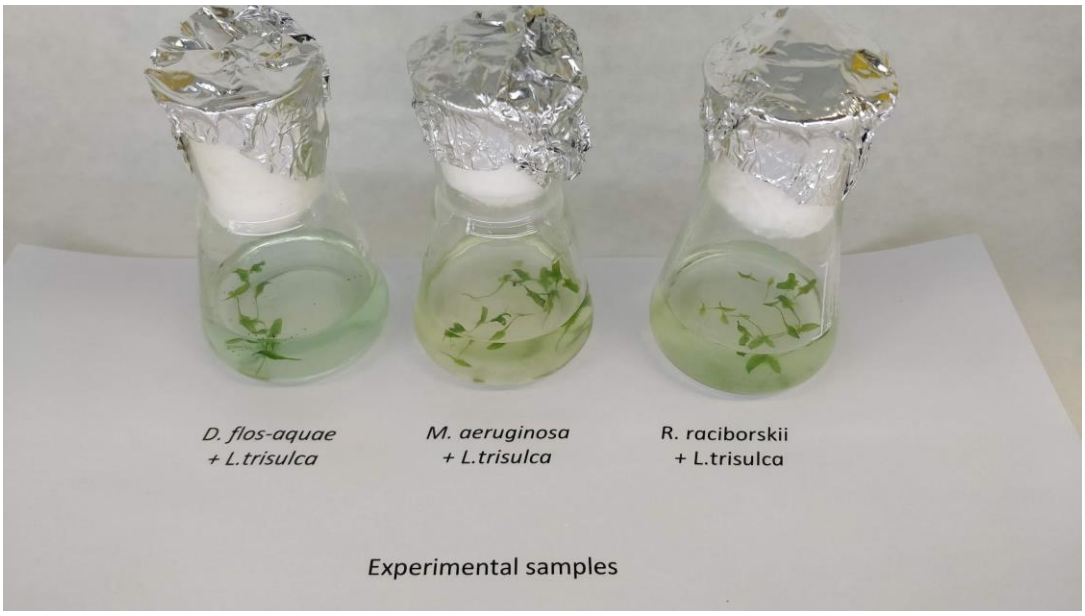

Figure S5. Illustrative photo showing co-cultivation of organisms

All 144 flasks (90 from the first and 54 from the second series) were randomly distributed in the phytotron and cultivated at 22±1°C with 80±5% humidity and 25 μmol photons $m^{-2}s^{-1}$ photosynthetically active radiation (PAR) under a 12-h photoperiod. All cultures were shaken daily.

Samples (flasks with cyanobacteria, plant, or pure medium) were collected at t_0 (initial day) and after 1, 4, 7, 11, and 14 days of cultivation. Every time, three independent replicates (three individual flasks) were sampled for each culture. Before proper analysis, all of the flasks were gently shaken for 10 min to mix the material.

Sample separation

medium

- right after medium collection, the pH and conductivity measurement,
- transfer of 5 mL to fresh tube and freeze for further ion concentration analysis,
- analysis of selected cations and anions on the DX600A ion chromatograph,
- transfer of one mL to HPLC vial and freeze for further toxin concentration analysis,

cyanobacterial cells

plant

- liquid nitrogen freezing,
- freeze-drying,
- dry weight (d.w.) determination

- adding 4 mL of 80% acetone and homogenization on an Omni Sonic Ruptor 400 homogenizer (3/8" processing tip, gradually increasing the power up to 400 watts, 10 seconds homogenization),
- microscopic verification of cells lysis completeness

- centrifugation for 5 min at 10.000 x g
- transfer 200 µL of the supernatant to a 96-well plate. Measurement of the absorbance at 470, 646 and 665 nm on a BioTek Synergy H1 microplate reader,
- transfer of 1 ml of the sample (in 80% acetone) to HPLC vial, evaporation to dryness and dissolving in 1 ml of 10% methanol

analysis of toxin concentration using Shimadzu Nexera-I LC-2040C 3D Plus UHPLC

Sample separation

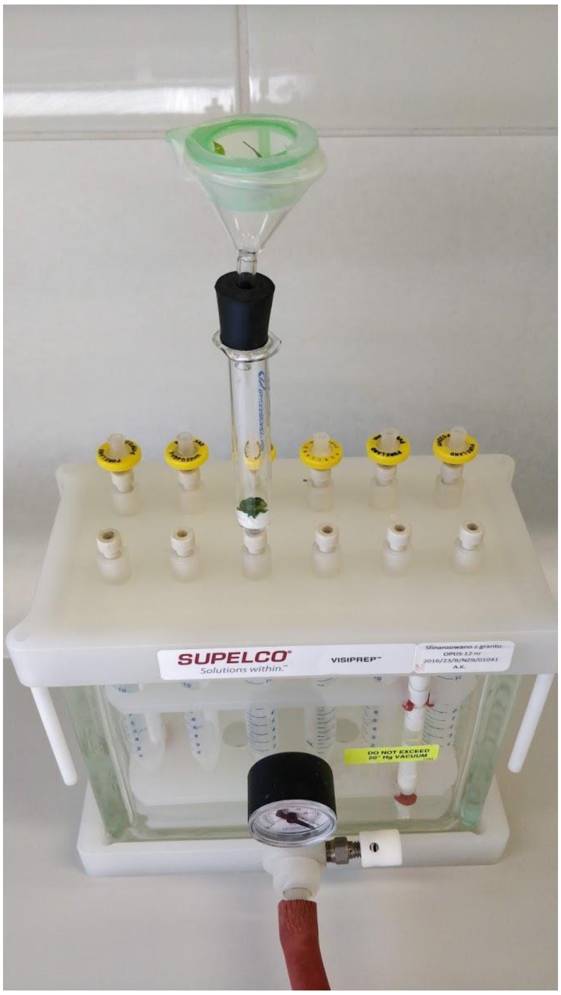

For each sample (flask), it was prepared column for separation. The cultures were poured into the separation columns. The macrophyte remained on the sieve, the cyanobacteria were retained on the microfiber filters, and the medium was collected in the tube

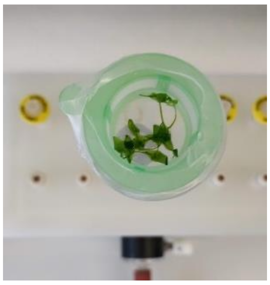

macrophyte on a plastic sieve

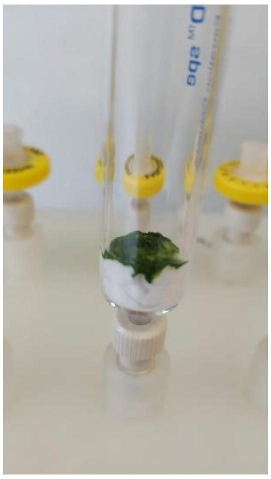

cyanobacteria cells on preweighed Whatman™ GF/C filter

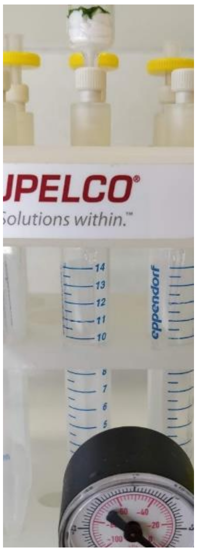

collected medium in tube

The total amount of cyanotoxins is given in the body of the manuscript. Here, it is additionally shown as content - µg per mg dry weight.

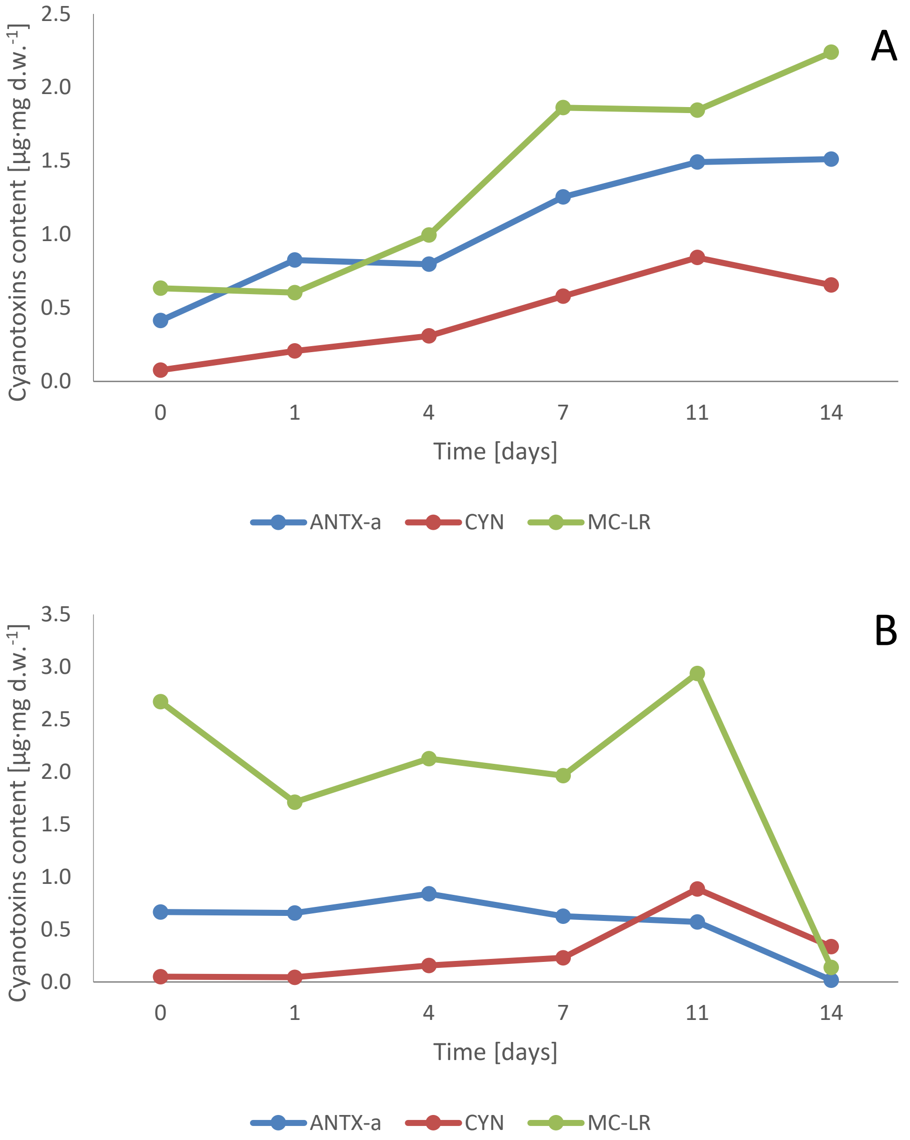

Figure S6. Cyanotoxins content calculated for mg of cyanobacterial dry weight within 14 days separately cultivation of *D. flos-aquae* (ANTX-a), *Raphidopsis raciborskii* (CYN), or *M. aeruginosa* (MC-LR) (A), or cyanobacteria co-cultivated with *L. trisulca* (B)
